# Supplementary material for: Substrate binding-induced conformational transitions in the omega-3 fatty acid transporter MFSD2A
Source: Nat Commun. 2023 Jun 9;14:3391. doi: 10.1038/s41467-023-39088-y (PMC10250862; doi:10.1038/s41467-023-39088-y)
Supplement: Supplementary file 1 — Supplementary Information [file 41467_2023_39088_MOESM1_ESM.pdf]

SUPPLEMENTARY INFORMATION

Substrate binding-induced conformational transitions in the omega-3 fatty acid transporter MFSD2A

Shana Bergman, Rosemary J. Cater, Ambrose Plante,
Filippo Mancia, and George Khelashvili

Supplementary Figures

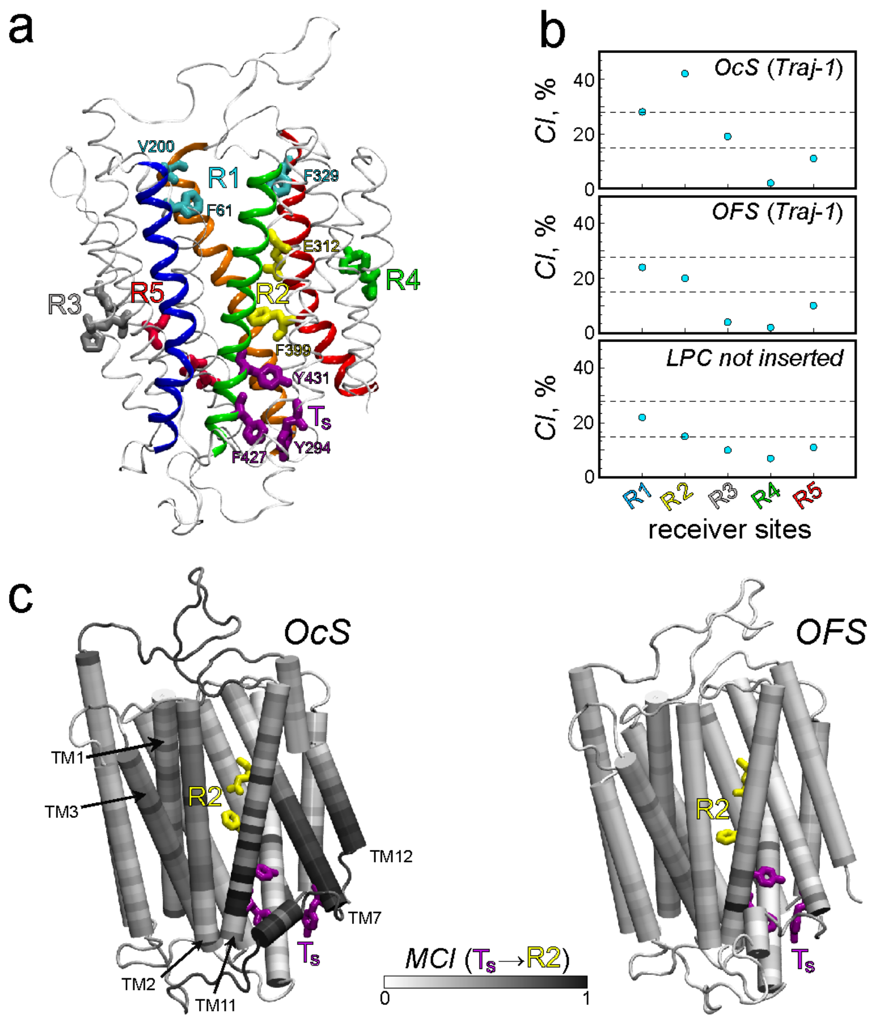

Supplementary Figure 1: Allosteric coupling between the IC ends of TM7/TM11 and the central region of ggMFSD2A. (**a**) Representative model of ggMFSD2A in the OFS highlighting the location of the *transmitter* (T_S) and *receiver* sites (R1-R5) used for *CI* calculations. The residues in each site are coloured according to the site label. The residues comprising T_S, R1, and R2 sites are labelled. (**b**) *CI* values calculated between the *transmitter* T_S site and the *receiver* sites R1-R5 in trajectories representing the OcS (top panel) and OFS (middle panel) ensembles (extracted from Traj-1, see Methods), as well as in a representative trajectory in which no substrate insertion occurred ("LPC not inserted"; bottom panel). Two horizontal lines demarcate regions of low, average, and high levels of coordination as obtained from the clustering of the *CI* data using the Fisher-Jenks algorithm (see Methods). (**c**) ggMFSD2A in OcS (left) and OFS (right) colored according to the normalized *MCI* values between the T_S and R2. Residues comprising these sites are highlighted as sticks and colored according to the site label. Selected TM helices are highlighted for reference.
