# Supplementary material for: Substrate binding-induced conformational transitions in the omega-3 fatty acid transporter MFSD2A
Source: Nat Commun. 2023 Jun 9;14:3391. doi: 10.1038/s41467-023-39088-y (PMC10250862; doi:10.1038/s41467-023-39088-y)
Supplement: Supplementary file 3 — Description of Additional Supplementary Files [file 41467_2023_39088_MOESM3_ESM.pdf]

File name: Supplementary Movie 1

Description: Traj-1 Molecular Dynamics trajectory showing spontaneous insertion of LPC-18:1 molecules via TM5/TM8 lateral opening into the central region of MFSD2A. The lysolipid is shown in surface representation. TMs 2, 5, 8, and 11 are shown in blue, orange, red, and green, respectively. TMs 7 and 10 are depicted in white. All the other segments of the protein are removed for clarity. Residue E312 (on TM7) in the central region, residue D92 (on TM2) in the primary Na^+ binding site, as well as the intracellular gate residues M182 (on TM5), F399 (on TM10), and W403 (on TM10) are all drawn as pink sticks. The Na^+ ion bridging the interactions between the inserted lysolipid and E312 is shown as yellow sphere. For this video, the trajectory was smoothened using the "smooth" VMD function applied to a window size of 8ns.

File name: Supplementary Movie 2

Description: Traj-2 Molecular Dynamics trajectory showing concomitant spontaneous insertion of two LPC-18:1 molecules via TM5/TM8 and TM2/TM11 lateral openings, respectively, into the central region of MFSD2A. The lysolipids are shown in surface representation. TMs 2, 5, 8, and 11 are shown in blue, orange, red, and green, respectively. TMs 7 and 10 are depicted in white. All the other segments of the protein are removed for clarity. Residue E312 (on TM7) in the central region, residue D92 (on TM2) in the primary Na^+ binding site, as well as the intracellular gate residues M182 (on TM5), F399 (on TM10), and W403 (on TM10) are all drawn as pink sticks. The Na^+ ion bridging the interactions between the lysolipid inserted via the TM5/TM8 opening and E312 is shown as yellow sphere. For this video, the trajectory was smoothened using the "smooth" VMD function applied to a window size of 8ns.
