# Supplementary material for: Substrate binding-induced conformational transitions in the omega-3 fatty acid transporter MFSD2A
Source: Nat Commun. 2023 Jun 9;14:3391. doi: 10.1038/s41467-023-39088-y (PMC10250862; doi:10.1038/s41467-023-39088-y)
Supplement: Supplementary file 6 — Reporting Summary [file 41467_2023_39088_MOESM6_ESM.pdf]

nature portfolio

Corresponding author(s): George Khelashvili

Last updated by author(s): 03/29/2023

Reporting Summary

Nature Portfolio wishes to improve the reproducibility of the work that we publish. This form provides structure for consistency and transparency in reporting. For further information on Nature Portfolio policies, see our Editorial Policies and the Editorial Policy Checklist.

Statistics

For all statistical analyses, confirm that the following items are present in the figure legend, table legend, main text, or Methods section.

n/a	Confirmed	
☐	☒	The exact sample size (*n*) for each experimental group/condition, given as a discrete number and unit of measurement
☐	☒	A statement on whether measurements were taken from distinct samples or whether the same sample was measured repeatedly
☒	☐	The statistical test(s) used AND whether they are one- or two-sided<br>*Only common tests should be described solely by name; describe more complex techniques in the Methods section.*
☒	☐	A description of all covariates tested
☒	☐	A description of any assumptions or corrections, such as tests of normality and adjustment for multiple comparisons
☒	☐	A full description of the statistical parameters including central tendency (e.g. means) or other basic estimates (e.g. regression coefficient) AND variation (e.g. standard deviation) or associated estimates of uncertainty (e.g. confidence intervals)
☒	☐	For null hypothesis testing, the test statistic (e.g. *F*, *t*, *r*) with confidence intervals, effect sizes, degrees of freedom and *P* value noted<br>*Give P values as exact values whenever suitable.*
☒	☐	For Bayesian analysis, information on the choice of priors and Markov chain Monte Carlo settings
☒	☐	For hierarchical and complex designs, identification of the appropriate level for tests and full reporting of outcomes
☒	☐	Estimates of effect sizes (e.g. Cohen's *d*, Pearson's *r*), indicating how they were calculated

Our web collection on statistics for biologists contains articles on many of the points above.

Software and code

Policy information about availability of computer code

Data collection	The reported MD simulations were carried out using NAMD 2.13 and OpenMM 7.4 software. Both are well-established, freely available software.
Data analysis	For all the analyses, except for Machine Learning-based and NbIT analyses, VMD 1.9.3 software was used which is well-established tool for data analysis and visualization and is freely available. The Machine Learning Deep Neural Network code is freely available via the following GitHub repository: https://github.com/weinsteinlab/DNN. The NbIT code is freely available via the following GitHub repository: https://github.com/weinsteinlab/NbIT. The DNN code implemented Keros and Tensorflow 2 libraries. For proline kink calculations, Prokink tool in Simulaid version 02/12/18 was used.

For manuscripts utilizing custom algorithms or software that are central to the research but not yet described in published literature, software must be made available to editors and reviewers. We strongly encourage code deposition in a community repository (e.g. GitHub). See the Nature Portfolio guidelines for submitting code & software for further information.

Data

Policy information about availability of data

All manuscripts must include a data availability statement. This statement should provide the following information, where applicable:

- Accession codes, unique identifiers, or web links for publicly available datasets
- A description of any restrictions on data availability
- For clinical datasets or third party data, please ensure that the statement adheres to our policy

The homology model of chicken MFSD2A in the outward facing state, as well as the trajectory files from Supplementary Movie 1 and Movie 2 are freely available via Zenodo public repository: https://zenodo.org/record/7933371#.ZF_jKOzMLQ0. Requests for all the additional structural models and MD simulation trajectories should be directed to and will be fulfilled by the lead contact. Any additional information required to re-analyze the data reported in this paper is available from the lead contact upon request.
The computational simulatoins described in this work were based on the following experimentally determined structures:
PDBID: 7N98, https://doi.org/10.2210/pdb7n98/pdb and PDBID: 7MJS, https://doi.org/10.2210/pdb7mjs/pdb

Human research participants

Policy information about studies involving human research participants and Sex and Gender in Research.

Reporting on sex and gender	N/A
Population characteristics	N/A
Recruitment	N/A
Ethics oversight	N/A

Note that full information on the approval of the study protocol must also be provided in the manuscript.

Field-specific reporting

Please select the one below that is the best fit for your research. If you are not sure, read the appropriate sections before making your selection.

☒ Life sciences ☐ Behavioural & social sciences ☐ Ecological, evolutionary & environmental sciences

For a reference copy of the document with all sections, see nature.com/documents/nr-reporting-summary-flat.pdf

Life sciences study design

All studies must disclose on these points even when the disclosure is negative.

Sample size	MD simulations for different constructs were carried out in multiple replicates as indicated in the manuscript. The number of replicates chosen and their timescales were sufficient in order to observe multiple events of substrate penetration into the transporter extracellular vestibule.
Data exclusions	No data, except for the initial minimization/equilibration phases described in the manuscript, were excluded from the analysis.
Replication	For each molecular construct, at least 24 independent replicates were considered.
Randomization	At each stage of MD simulations, the velocities of atoms in the system were randomized.
Blinding	Data was not blinded

Reporting for specific materials, systems and methods

We require information from authors about some types of materials, experimental systems and methods used in many studies. Here, indicate whether each material, system or method listed is relevant to your study. If you are not sure if a list item applies to your research, read the appropriate section before selecting a response.

Materials & experimental systems

n/a	Involved in the study
☒	☐ Antibodies
☒	☐ Eukaryotic cell lines
☒	☐ Palaeontology and archaeology
☒	☐ Animals and other organisms
☒	☐ Clinical data
☒	☐ Dual use research of concern

Methods

n/a	Involved in the study
☒	☐ ChIP-seq
☒	☐ Flow cytometry
☒	☐ MRI-based neuroimaging
